# Complete Genome Sequence of a Recent Panzootic Virulent Newcastle Disease Virus from Pakistan

**DOI:** 10.1128/genomeA.00658-15

**Published:** 2015-06-18

**Authors:** Abdul Wajid, Muhammad Wasim, Shafqat F. Rehmani, Tasra Bibi, Nazir Ahmed, Claudio L. Afonso

**Affiliations:** a Quality Operations Laboratory (QOL), University of Veterinary and Animal Sciences, Lahore, Pakistan; b Institute of Biochemistry and Biotechnology (IBBt), University of Veterinary and Animal Sciences, Lahore, Pakistan; c Karakoram International University, Gilgit, Pakistan; d Exotic and Emerging Avian Disease Research Unit, Southeast Poultry Research Laboratory, Agricultural Research Service, USDA, Athens, Georgia, USA

## Abstract

The genome sequence of a new strain of Newcastle disease virus (NDV) (chicken/Pak/Quality Operations Lab/SFR-611/13) is reported here. The strain was isolated from a vaccinated chicken flock in Pakistan in 2013 and has panzootic features. The genome is 15,192 nucleotides in length and is classified in subgenotype VIIi of genotype VII, class II.

## GENOME ANNOUNCEMENT

Newcastle disease (ND) is caused by an avian paramyxovirus of serotype 1 (APMV-1), and it is listed as a serious disease by the World Organization for Animal Health, affecting >236 avian species with wide geographical distribution (1). APMV-1, also designated Newcastle disease virus (NDV), belongs to the genus *Avulavirus* of the *Paramyxoviridae* family, and it has an enveloped, nonsegmented, and negative-sense RNA genome with six transcriptional units (3′-NP-P-M-F-HN-L-5′). Phylogenetic analysis of isolates of the virus worldwide has identified large genetic variability, and at the moment, there are up to 18 different genotypes (2, 3). Recently a new subgenotype of NDV (VIIi) with epizootic characteristics has been identified and characterized (4, 5). Viruses of this genotype have rapidly disseminated from Indonesia, to Pakistan, the Middle East, Europe, and Africa. The 2013 isolate described here is part of this lineage and is important because it has been found to be present in at least 20 Newcastle disease-vaccinated chicken farms in Pakistan, suggesting unique virulence and a capacity to disseminate (4, 5).

The virus was isolated and propagated in 9-day-old chicken embryonated eggs from a flock free from NDV antibody. Viral RNA was isolated from the allantoic fluids using TRIzol LS (Invitrogen, USA), and cDNA was synthesized using the random hexamer primer with the Transcriptor first-strand cDNA synthesis kit (Fermentas, USA), per the manufacturer’s recommendations. The complete genome sequence was obtained using an overlapping real-time PCR (RT-PCR) strategy with 22 different primers pairs using Platinum PCR SuperMix high-fidelity polymerase (Invitrogen) that included the ends. The products were purified using the GeneJET gel extraction kit (Fermentas) and cloned into the TOPO TA vector (Invitrogen) accordingly. For sequencing, an ABI 3130 automated sequencer (ABI, Inc., Foster City, CA) was used, and for editing and assembly, the BioEdit software was used (6). Sequence analysis revealed that the full-genome length of the isolated strain currently designated chicken/Pak/Quality Operations Lab/SFR-611/13 is 15,192 nucleotides (nt). Phylogenetic analysis of the complete genome classified this strain into class II, genotype VII, subgenotype VIIi. The nucleotide sequence had the highest homology (99%) with the sequence of Indonesian strain chicken/Banjarmasin/010/10 (GenBank accession no. HQ697254).

The strain chicken/Pak/Quality Operations Lab/SFR-611/13 has a virulent pathotype, with ^112^RRQKR^116^ at the C terminus of the F2 protein and F at residue 117. The data presented here provide evidence of a novel strain of NDV in the region, and understanding the prevalence and variation of newly emerging NDV strains has great significance for the prevention and control of ND.

### Nucleotide sequence accession number.

The complete genome sequence of the newly emerging NDV strain chicken/Pak/Quality Operations Lab/SFR-611/13 has been deposited in GenBank under the accession no. KM670337.
